# Does Language Matter? Exploring Chinese–Korean Differences in Holistic Perception

**DOI:** 10.3389/fpsyg.2016.01508

**Published:** 2016-10-17

**Authors:** Ann K. Rhode, Benjamin G. Voyer, Ilka H. Gleibs

**Affiliations:** ^1^Department of Marketing, ESCP EuropeParis, France; ^2^Ecole de Management de la Sorbonne (EMS), Université Paris 1 Panthéon-SorbonneParis, France; ^3^Department of Marketing, ESCP EuropeLondon, UK; ^4^Department of Psychological and Behavioural Science, London School of Economics and Political ScienceLondon, UK

**Keywords:** culture, attention, language, thinking for speaking, linguistic relativity, replication

## Abstract

Cross-cultural research suggests that East Asians display a holistic attentional bias by paying attention to the entire field and to relationships between objects, whereas Westerners pay attention primarily to salient objects, displaying an analytic attentional bias. The assumption of a universal pan-Asian holistic attentional bias has recently been challenged in experimental research involving Japanese and Chinese participants, which suggests that linguistic factors may contribute to the formation of East Asians' holistic attentional patterns. The present experimental research explores differences in attention and information processing styles between Korean and Chinese speakers, who have been assumed to display the same attentional bias due to cultural commonalities. We hypothesize that the specific structure of the Korean language predisposes speakers to pay more attention to ground information than to figure information, thus leading to a stronger holistic attentional bias compared to Chinese speakers. Findings of the present research comparing different groups of English, Chinese, and Korean speakers provide further evidence for differences in East Asians' holistic attentional bias, which may be due to the influence of language. Furthermore, we also extend prior theorizing by discussing the potential impact of other cultural factors. In line with critical voices calling for more research investigating differences between cultures that are assumed to be culturally similar, we highlight important avenues for future studies exploring the language-culture relationship.

## Introduction

The relationship between language and culture and their influence on cognition and perception has been a major issue of concern for psychologists, anthropologists, and philosophers alike since Boas, Sapir, and Whorf theorized that language influences, or might even determine, the way we see the world (see Pinker, 1994; Levinson, 2003; Casasanto, 2008 for a review). While Boas and Sapir suggested that culture influences language but not vice versa, Whorf was the first to suggest that language and culture may interact, and that language might play a larger role in the context of long historical interaction than assumed (Lucy, 1992). For centuries the debate on the relationship between language and culture remained mainly focused on the direction of a potential causal relationship, and since researchers used to take mutually exclusive positions, the controversial issue remained unsolved. More recently, research investigating the evolution of language and culture led to new insights and to a revival of the old debate (e.g., Christiansen and Chater, 2008; Richerson and Boyd, 2010). While there is a consensus among scientists that language is the result of a gene-culture coevolution (e.g., Pinker and Bloom, 1990; Feldman and Laland, 1996; Nettle, 2007), evolutionary linguists differ greatly on the details of this coevolution. While some authors argue that human language is a complex biological adaptation which evolved by natural selection (Pinker, 2003), others hold the view that culture played a large role in adapting language to pre-linguistic capacities (e.g., Tomasello, 2008; Kirby et al., 2009). In addition, ample research provides evidence that both cultural and linguistic factors influence to some extent perception, information processing, and cognition. However, it remains unclear to what extent culture and language may interact. Enfield (2012) points out that research in this area is particularly difficult, as expertise in both linguistics and anthropology is required, and fundamental questions, such as “How to define culture? Is “culture” even a useful concept? Is it possible to distinguish culture from language? If so, how to make the distinction and how to build a logical argument that the two are related?” (p. 160) remain unanswered.

The present research aims at contributing to the debate about the extent to which language and culture may interact and penetrate core areas of perception, information processing, and cognition (e.g., Gumperz and Levinson, 1991; Hunt and Agnoli, 1991; Hardin and Banaji, 1993). It also responds to calls for more replications of novel findings in the light of the crisis of confidence in psychological science, which has raised questions about the trustworthiness of research findings (Pashler and Wagenmakers, 2012; Klein et al., 2014; Earp and Trafimow, 2015). While psychologists debate on the question of promoting “direct” vs. “conceptual” replications, findings of efforts to conduct “exact” or “direct” replications of important papers are mixed (e.g., Cesario, 2014; Simons, 2014). Critical voices argue that exact replication is impossible, since even if one used the exact same procedure, participants change over time (Stroebe and Strack, 2014). In addition, contextual factors, so-called “hidden moderators,” are likely to affect the results of direct replications. Recent research suggests that contextual factors (e.g., time, location, culture) are associated with reproducibility, even after adjusting for methodological variables of the original research that are linked to replication success (Van Bavel et al., 2016). Thus, some scholars argue for increasing the number of “conceptual replications” and “replications with extensions” that provide better evidence of the external validity of published findings than direct replications (Lynch et al., 2015). According to Locke (2015), “replications with variation” (i.e., conceptual replication) can also contribute to theory building, as many varied studies are necessary to develop and refine a core idea.

Besides conceptually replicating Tajima and Duffield's (2012) research we highlight potential avenues for future cross-cultural research exploring the language-culture relationship. Findings of our experimental study involving English, Chinese, and Korean speakers, support Tajima and Duffield's (2012) research that challenges the assumption of a pan-Asian holistic attentional bias resulting from socio-cultural influence only. While the observed effect may be due to the influence of language as hypothesized, we also discuss the potential role of other cultural factors and alternative explanations.

## Theoretical background

### Language, culture, and attention

A growing body of research in the field of cognitive (neuro)science provides evidence for the influence of language on human perception (e.g., Meteyard et al., 2007). Research findings suggest a direct influence of language on early visual perception and a close integration of conceptual and perceptual systems, supporting theories of embodied cognition (e.g., Wilson, 2002; Borghi and Pecher, 2012), and grounded cognition (Barsalou, 2008).

A separate stream of research in the field of cross-cultural psychology explores the influence of cultural factors on perception. The vast majority of research uses the dimension of individualism–collectivism to operationalize culture and explores foremost Western–East Asian differences (e.g., Markus and Kitayama, 1991; Oyserman et al., 2002; Kastanakis and Voyer, 2014). Pioneering research by Masuda and Nisbett (2001) indicates that Westerners pay attention primarily to salient objects, displaying an analytic attentional bias, whereas East Asians display a holistic attentional bias by paying attention to the entire field and to relationships between objects and the field. More recent research using different methods support these findings (see Boland et al., 2008; Han and Ma, 2014 for an overview). For instance, eye-tracking research by Chua et al. (2005) revealed that North Americans fixate more on focal objects of pictures than Chinese. In contrast, Chinese make more saccades to the background than North Americans.

Nisbett et al. (2001) reason that differences in attention might result from long-term cultural differences that are rooted in differing social structures and intellectual traditions of ancient Greece and ancient China. Greek intellectual traditions can be described as analytic, since the attentional focus is on some salient object, which is detached from its context, assessed in terms of its attributes and assigned to a category in order to find out the rules that govern its behavior. In contrast, intellectual traditions in ancient China, which have been shaped by Confucianism, Taoism, and Buddhism, are holistic in nature and might have led to the development of East Asians' focus on relationships between objects and the field, and to the tendency of explaining events on the basis of these relationships. Differences in the development of science, mathematics, and philosophy reflect this cultural dichotomy between East Asia and the West (e.g., Nakamura, 1964; Ji et al., 2001; Nisbett, 2003).

Moreover, Nisbett and Masuda (2003) hypothesize that living in the complex, interdependent ancient Chinese society, which emphasized social harmony and role relations, might have fostered holistic perception and cognition. In contrast, the less complex and less role-constraint society of ancient Greece, which allowed people to develop a sense of personal agency, might have fostered analytic perception and cognition. Studies involving collectivistic non-Asian samples (e.g., Italians, Croatians) and samples of different groups belonging to one collectivistic culture (e.g., farming communities and herding communities in Turkey) provided evidence for Nisbett and Masuda's (2003) assumption that interdependent social structures might have contributed to the emergence of the holistic attentional bias (Knight and Nisbett, 2007; Uskul et al., 2008; Varnum et al., 2008). More recent research investigates how cross-cultural differences in attention may be sustained over generations. Senzaki et al. (2016) provide first empirical evidence that children learn at a young age culturally dominant modes of attention from their parents, who communicate with them either in an object-oriented mode or in a context-sensitive mode.

### “Thinking for speaking” effects

However, more recent experimental research by Tajima and Duffield (2012), comparing Japanese and Chinese participants' attentional patterns, challenges the assumption of a universal pan-Asian holistic attentional bias. English, Japanese, and Chinese participants were asked to complete picture description tasks and recall tasks about contextual information (ground information). Results of the tasks provided evidence for the authors' hypothesis that the holistic attentional bias of Japanese participants might be reinforced through the impact of language. In the description tasks, Japanese participants reported significantly more ground information overall and they mentioned ground information before figure information (salient objects) more often than Chinese and English participants. Results of the recall tasks also indicated that Japanese remembered ground information significantly better than Chinese and English participants. Drawing on Slobin's (1991, 1996) Thinking for Speaking hypothesis, Tajima and Duffield (2012) conclude that the specific structure of the Japanese language may predispose Japanese speakers to pay more attention to ground information than to figure information.

In the context of the Sapir–Whorf hypothesis, which makes assumptions about the impact of language on cognition, Slobin's (1991) Thinking for Speaking hypothesis is ascribed to the theoretical framework of Linguistic Relativity (e.g., Franks, 2011). In contrast to the doctrine of Linguistic Determinism, which postulates that language determines the way we think and perceive the world, theories of Linguistic Relativity take the less deterministic approach that culture—through language—influences thought, and that specifically the formal structures of language affect the way we think (Whorf, 1956; Gumperz and Levinson, 1996; Lucy, 1996). Based on findings of a number of cross-linguistic studies in different cultures, Slobin (1991, 1996) hypothesized that rhetorical styles of different languages “reflect different patterns of thinking for speaking—different on-line organization of the flow of information and attention to the particular details that receive linguistic expression” (Slobin, 1991, p. 14). This idea has been further developed in a specific mode of cognition, called “Thinking for Speaking.”

In the evanescent time frame of constructing utterances in discourse one fits one's thoughts into available linguistic frames. “Thinking for speaking” involves picking those characteristics of objects and events that (a) fit some conceptualization of the event, and (b) are readily encodable in the language (Slobin, 1996, p. 76).

Moreover, Slobin (1991) speculated that thinking for speaking effects might predispose speakers to develop—through habituation—particular attentional patterns which might even exist outside of linguistic contexts. For instance, the linguistically encoded honorific systems of the Korean and the Japanese language might require the speaker to pay more attention to status relations between individuals than speakers of languages without an honorific system. Hence a Korean or Japanese speaker might develop the habit of attending more to status relations and relationships, even in non-linguistic contexts (Tajima and Duffield, 2012).

### The specificity of the korean language

A comprehensive literature review revealed that—except for Tajima and Duffield's (2012) research—prior studies investigated cross-cultural differences in visual attention comparing solely one East Asian sample with one Western sample. East Asian samples of these studies consist either of Chinese speakers, Japanese speakers, Asian Americans, or a “mix” of East Asians of different nationalities (see Table 1 for an overview). Critical voices claim that the dominance of two-country comparisons is a major methodological concern of current cross-cultural research. Comparing two countries does not allow us to rule out the influence of other factors (e.g., linguistics) that may account for differences between the two cultures (Varnum et al., 2010; Engelen and Brettel, 2011). In addition, it is easy to take findings that document differences between two distant cultures and to overgeneralize those findings to groups that are classified as culturally similar. However, this practice can lead to stereotypes of cultural differences that may not be true (Matsumoto and Jones, 2009).

**Table 1:** **Cross-cultural research investigating differences in visual attention**.

**Publication Title**	**Samples**	**Methods/Measures**
Chua et al., 2005	European Americans, Chinese	Eye tracking
Fong et al., 2014	European Americans, East-Asian Americans	ERP
Goh et al., 2007	US-Americans, Singaporeans	fMRI
Goto et al., 2010	European Americans, East Asian Americans	ERP
Goto et al., 2013	European Americans, Asian Americans	ERP
Gutchess et al., 2006	Americans, East Asians	fMRI
Ji et al., 2000	Study 1a and 1b: Taiwanese, Caucasian US-Americans	Experiments
	Study 2: European Americans, East Asians (mainly from China, Korea, Japan)	
Kitayama et al., 2003	Japanese, US-Americans	Experiments
Knight and Nisbett, 2007	Northern Italians, Southern Italians	Experiments
Lewis et al., 2008	European Americans, East Asian Americans of Chinese, Korean and Japanese descent	ERP
Masuda et al., 2008a	Study 1: None	Experiments
	Study 2: Americans (Caucasians, African Americans), East Asians (Taiwanese, Koreans, Japanese, Chinese)	
	Study 3: Americans (Westerners, Asian Americans), Japanese	
Masuda et al., 2008b	Study 1: Americans (Anglophones), Japanese	Experiment, eye tracking
	Study 2: Anglophone Westerners (from Australia, Canada, New Zealand, UK, USA), Japanese	
Masuda and Nisbett, 2001	Study 1: Americans, Japanese Study 2: Americans, Japanese	Experiments
Masuda and Nisbett, 2006	Study 1: Americans, East Asians (Chinese, Japanese, Koreans) Study 2: Americans, Japanese Study 3: Americans, Japanese	Experiments
Miyamoto et al., 2006	Study 1: Americans, East Asians Study 2: Americans, Japanese	Experiments
Miyamoto and Wilken, 2010	Study 1: European Americans, Japanese Study 2: European Americans, Japanese	Experiments
Oishi et al., 2014	Study 1: Americans, Japanese, Argentinians Study 2: Americans, Japanese Study 3: Americans, Japanese	Experiments
Rayner et al., 2007	Americans, Chinese, English-Chinese bilinguals (Americans of Chinese descent)	Eye tracking
Russell et al., 2015	European Canadians, Japanese	ERP
Senzaki et al., 2014	Study 1: European Canadians, Japanese Study 2: European Canadians, Japanese	Eye tracking
Senzaki et al., 2016	Study 1: Canadian children (European-Canadian, Hispanic, African-Canadian, and mixed ethnicity), Japanese children	Experiments
	Study 2: European-Canadian parent-child dyads, Japanese parent-child dyads	
Tajima and Duffield, 2012	Study 1: Japanese, Chinese, British	Experiments
	Study 2: Japanese, Chinese, British	
Uskul et al., 2008	Turkish (farmers, fishermen, herders)	Experiments
Varnum et al., 2008	Study 1: Western Europeans, Central and Eastern Europeans	Experiments
	Study 2: Americans, Croats	

For several decades of cross-cultural research identifying clusters of culturally similar societies, Korea is mentioned due to its Confucian heritage in the same breath as China, Taiwan, Hong Kong, Singapore, and Japan (e.g., Gupta et al., 2002). However, to the best of our knowledge, no cross-cultural study to date has investigated the holistic attentional bias among Korean native speakers. In this regard, the purpose of the present research is three-fold: (i) investigating the holistic attentional bias for the first time by looking at a sample of native Korean speakers; (ii) conducting a three-country comparison including two cultures that are commonly classified as culturally similar; and (iii) extending and conceptually replicating Tajima and Duffield's (2012) research in order to investigate the scope of a “pan-Asian” holistic attentional bias. We examine differences in holistic attentional patterns further by comparing Mandarin Chinese with Korean—a language that is similar to Japanese in terms of semantical and syntactical elements, but that does not belong to the Japonic language family (e.g., Vovin, 2010). Despite similar grammatical features and some overlap in vocabulary, Japanese and Korean are classified by linguists as language isolates (Whitman, 2012).

Korean and Japanese stand in contrast to English and Mandarin Chinese both in terms of semantically and syntactically central elements. According to Talmy (2000, p. 334), “the Figure has syntactic precedence over the Ground” in English and both the physical location of events and the temporal ordering of events follow figure information. In Mandarin Chinese, the figure has usually, but not always precedence over the ground (Tai, 1985; Tajima and Duffield, 2012). In contrast to the figure-to-ground-order in English and Chinese sentences, Korean sentences show mainly ground-to-figure-order (Tajima and Duffield, 2012). In cases where Korean sentences take a figure-to-ground-order, topic constituents (in Korean 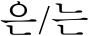, eun/neun) presenting ground information and preceding the subject, are usually placed at the beginning of the sentence to establish the context (e.g., Sohn, 2001). Korean, Mandarin Chinese, and English also differ syntactically in terms of phrasal constituents. In head-initial languages, such as English, the verb precedes the direct object (sentence structure: subject-verb-object). In head-final languages however, such as Korean, the object precedes the verb (sentence structure: subject-object-verb; e.g., Lee and Ramsey, 2000; Tajima and Duffield, 2012). Mandarin Chinese shows mainly the order of head-initial languages and in some cases the order of head-final languages (Huang, 1994).

Moreover, Korean politeness conventions might reinforce ground-to-figure-order in sentences. In some East Asian languages, such as in Korean and Japanese, “The more contextual (Ground) information is mentioned before Figure information, the more polite the utterance is perceived to be” (Tajima and Duffield, 2012, p. 686). As Tajima and Duffield (2012) note, starting utterances with the main point without first establishing background reference is often interpreted as impatience, rudeness, or arrogance. While English speakers can skip for instance ground information to focus on figure information, skipping ground information in favor of figure information would often violate Korean politeness conventions.

Drawing on the Thinking for Speaking hypothesis (Slobin, 1991), we hypothesize that Korean speakers' linguistically-formed habit of placing ground information before figure information might lead to the tendency of paying more attention to ground information (the field) than to figure information (salient objects). In line with Tajima and Duffield's (2012) research, we anticipate a split holistic attentional bias among East Asians and a linear effect of language on the amount of pieces of ground information: Korean participants should report the highest overall number of ground information, followed by Chinese participants, and then English participants. A similar linear effect is anticipated for the recall task, which tests participants' ability to remember ground information of the presented pictures.

## Materials and methods

### Participants

Sample size was estimated following Cohen's (1988) guidelines by using G^*^Power 3. We calculated a sample size that allows to detect large effect sizes (*f* = 0.40) in an analysis of covariance (e.g., Faul et al., 2007). Parameters used to calculate the sample size estimate (*N*_*estimate*_ = 64) were statistical power (set to 0.80) and type I error level (set to 0.05). Based on this estimate, 90 monolingual (30 English, 30 Chinese, and 30 Korean speakers) participants (*N* = 90; age: *M* = 27.9; 58 women), all residing in major cities in their home country (Britain, China, South Korea), contributed in their native language to an online study. None of the English participants had learned or were learning an East Asian language at the time of study. In terms of the highest completed level of education, 11 participants reported having a high school diploma, while all other participants reported having a university degree (Bachelor, Master, Doctorate).

### Ethics statement

The research project received approval from the third author's institution research ethics committee on 27th February 2014. All participants were informed about the purpose of the study and the anonymization of all data, including demographic information about gender, nationality, educational background, and native language.

### Materials

Participants were presented with an adapted online version of Tajima and Duffield's (2012) questionnaire consisting of two tasks: a *description task* and a *recall task*. Questionnaires were prepared by bilinguals using the method of back-translation (Brislin, 1986) from English into Korean and Mandarin Chinese. Since the original study was paper-based, we conducted a pre-test of the online study. The overall number of stimuli was reduced by half, since a large number of participants dropped out of the pre-test stating that it took long (i.e., more than 30 min) to complete the questionnaire. Thus, the *description task* and the *recall task* involved three instead of six pictures.

### Procedure

After answering demographic questions (nationality, gender, educational background, native language, proficiency in other languages etc.), participants completed two tasks: a *description task* and *a recall task* consisting of two separate parts. In the description task, participants were presented with pictures and were asked to provide a written description of five to six sentences in their native language, using freely both figure and ground information (i.e., references to place, time, field, or inferred antecedent events). Upon completion of the picture description task, the two parts of the recall task were presented separately. In the first part of the recall task, participants were presented with a set of picture fragments and had to identify which fragments were belonging to the pictures they had seen. In the second part of the recall task, participants were asked to answer questions about specific ground information of the pictures they had seen.

### Data analysis

Participants' responses to the picture description task were coded to identify the type of ground information (i.e., place, time, field or inferred antecedent events, or situations) and figure information according to Tajima and Duffield's (2012) data coding scheme. Two Korean-English bilinguals and two Chinese native speakers who were blind to the hypotheses independently coded the data in the original languages. Intercoder agreements on the coded number of ground information were high [ICC(3, 2)_*English*_ = 0.97, ICC(3, 2)_*Chinese*_ = 0.95, ICC(3, 2)_*Korean*_ = 0.96], and disagreements were resolved in discussions between the coders and the first author. The number of ground information of each picture, the overall number of ground information in the picture description task and the total number of correct answers of the recall task were used for a statistical analysis.

### Results

**Figure 1:**
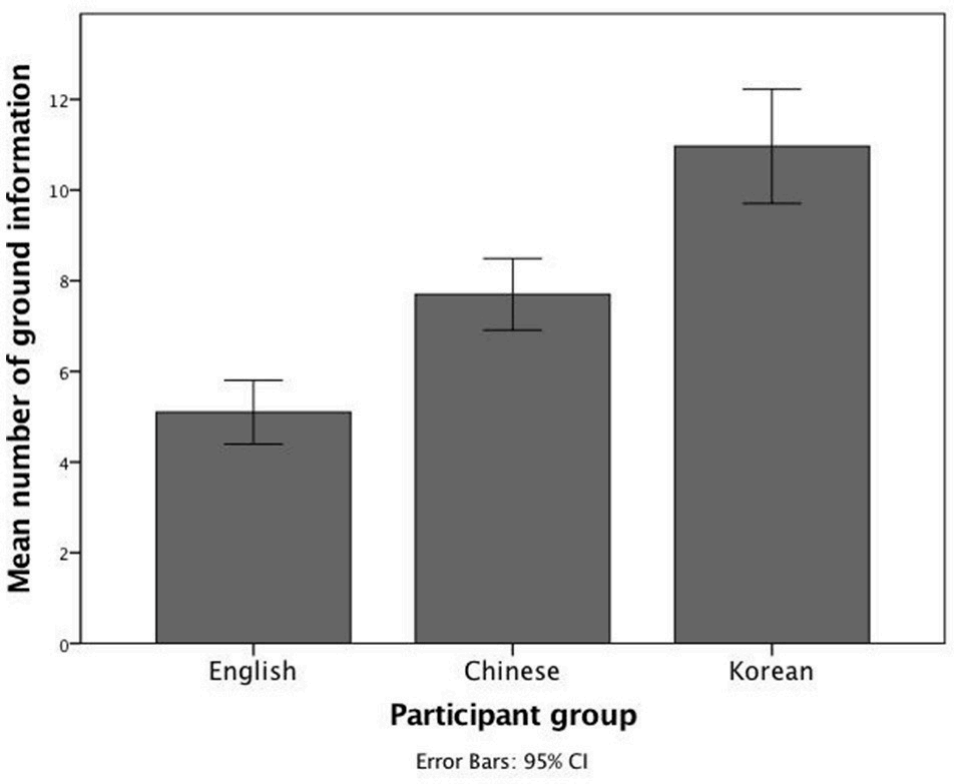
**Bar diagram showing the mean number of ground information mentioned by English, Chinese, and Korean participants in the picture description task**. Korean participants reported the highest overall number of ground information, followed by Chinese participants, and English participants.

As hypothesized, Korean speakers generally placed ground information ahead of figure information in their sentences and mentioned more ground information overall than English and Chinese speakers. An ANOVA looking at the effect of language on the number of ground information revealed significant differences between the participant groups in the *picture description task* [*M*_*Koreans*_ = 10.97, *SD* = 3.38; *M*_*Chinese*_ = 7.70, *SD* = 2.12; *M*_*English*_ = 5.10, *SD* = 1.88; *F*_(2, 87)_ = 39.99, *p* < 0.001 and η^2^ = 0.48]. Similar to the results of the research conducted by Tajima and Duffield (2012), moderate to large effects were found in all three group comparisons, with the largest effect being between Korean and English participants (Cohen's *d* = −2.15) followed by the effect between English and Chinese participants (Cohen's *d* = −1.30) and the one between Koreans and Chinese (Cohen's *d* = −1.16). In sum, Korean participants reported the highest overall number of ground information, followed by Chinese participants, and English ones (Figure 1). In the *recall task* however, the ANOVA did not reveal any significant differences between the participant groups [*M*_*Koreans*_ = 6.33, *SD* = 1.35; *M*_*Chinese*_ = 5.73, *SD* = 1.96; *M*_*English*_ = 5.90, *SD* = 1.73; *F*_(2, 87)_ = 0.997, *p* = 0.37 and η^2^ = 0.02]. A summary of findings is presented in Table 2.

**Table 2:** **Summary of findings**.

**Picture description task**		**Recall task**	
***F*_(2, 87)_ = 39.99, *p* < 0.001, η^2^ = 0.48**		***F*_(2, 87)_ = 0.997, *p* = 0.37, η^2^ = 0.02**	
*M_Koreans_* = 10.97	*SD* = 3.38	*M_Koreans_* = 6.33	*SD* = 1.35
*M_Chinese_* = 7.70	*SD* = 2.12	*M_Chinese_* = 5.73	*SD* = 1.96
*M_English_* = 5.10	*SD* = 1.88	*M_English_* = 5.90	*SD* = 1.73

## Discussion

### Implications of the findings

This first conceptual replication study of Tajima and Duffield's (2012) provides further evidence that challenges the assumption of a pan-Asian holistic bias. Findings of the description task suggest that language may have an effect on visual attention in linguistic contexts. However, no effect was observed in the recall task. On the one hand, the absence of effect in the recall task condition could be explained by methodological issues, such as the reduction of the number of stimuli compared with the original study. On the other hand, these results may support prior experimental research that found little to no effects of cross-linguistic differences on memory (e.g., Gennari et al., 2002; Papafragou et al., 2002).

The present research contributes to the literature in three ways. First, it supports other studies indicating that language may influence the perception of visual stimuli, causing speakers of different languages to attend to aspects of a scene that are particularly encoded or marked in their native language (e.g., Altmann and Kamide, 2004; Bock et al., 2004; Lai et al., 2013). For instance, research by Senzaki et al. (2014) demonstrates that cultural variations in patterns of attention do not arise when participants simply observe visual information, but when they construct narratives of their observations. As Papafragou et al. (2008) point out, preparing for language production might have rapid effects on how speakers of different languages allocate visual attention to components of a scene, such as in the case of motion events. If people need to talk about what they see, they are likely to shift their attention focus toward aspects of the scene that are relevant for purposes of sentence planning—which has been described by Slobin (1991, 1996) as thinking for speaking effects. In addition, our study is in line with other studies suggesting that verbal information processing and visual information processing are closely connected, and that the activation of one system may have an impact on the other (Hong et al., 2000; Fausey et al., 2010).

Second, this study provides further evidence challenging the assumption of a universal pan-Asian holistic attentional bias, which has been assumed to result from socio-cultural impact only (i.e., interdependent social structures and holistic intellectual traditions). Instead, a more complex picture emerges where structures of specific languages, which emphasize contextual information (ground information), may reinforce socio-culturally induced holistic attentional patterns. Taken together with findings that different ecocultural groups, which belong to the same collectivistic culture, display holistic attentional patterns of different magnitudes (Uskul et al., 2008), the present research also suggests that analytic and holistic attentional patterns might relate to each other on a continuum.

Third, results contribute to the literature by providing evidence of the interactional relationship between language and culture and their potentially joint impact on particular domains of perception and cognition. This is in line with prior studies by Ishii et al. (2003) and Ji et al. (2004) indicating that culture and language interactively influence cognitive processes. Following Franks (2011, p. 312), “[l]anguage may both be a vehicle for cultural influences and a discrete influence that is separable from culture.”

### Limitations

The present study has four main limitations. First, while drawing on visual stimuli adapted from Tajima and Duffield's (2012) study, the number of stimuli in the recall task was reduced and the time that participants spent on the description task was neither controlled nor limited (constraints of online surveys: Buchanan and Smith, 1999). This could explain why results from the recall task were inconclusive.

Second, it cannot be ruled out that Korean participants were less field-oriented than Chinese participants and simply more meticulous when completing the experimental tasks. By explicitly asking participants to report both background information as well as details about focal objects, it could be investigated whether Korean and Chinese participants differ in terms of reporting background information and details about focal objects, or merely with regard to reporting background information.

Third, the present research was conducted at the level of explicit cognition, and therefore cannot be conclusive about the way Korean speakers or Chinese speakers experience the world or how they think about it at large. As Slobin (1991) points out, every language is “a subjective orientation to the world of human experience, and this orientation *affects the ways in which we think while we are speaking*” (Slobin, 1991, p. 23) While languages are highly selective schematic maps, they are not exact representations of our experience or of our thought (Slobin, 1996; Clark, 2003).

Fourth, it is possible that not language, but other moderators of cultural differences are responsible for the observed effect, such as differences in Chinese and Korean participants' sense of agency (Miyamoto and Ji, 2011; Miyamoto, 2013). In addition, societal and economic changes caused by globalization are likely to foster independence and to reduce interdependence in many societies (Varnum et al., 2010). This is particularly the case in China (e.g., Yan, 2010). Research by Russell et al. (2015), Fong et al. (2014), and Goto et al. (2010, 2013) suggests that independent self-construal orientation is associated with greater context independent, analytic semantic processing styles. Thus, it should be noted that one cannot rule out that differences in self-construal orientation may (at least partly) account for differing magnitudes of holistic attentional bias in the two Asian samples.

Most importantly, we do not seek to refute prior findings documenting across a wide range of studies the influence of culture on visual attention. Based on the findings of the present study, we hope to stimulate further research that goes beyond the common comparison of two distant cultures and that takes into account the potential influence of language.

### Future research

To better understand the effect of language on visual attention and information recollection, the next steps of this research should involve a series of replications including (i) the use of a larger number of stimuli in the recall task and a time limit in the description task; (ii) the use of a self-construal scale (e.g., Singelis, 1994) to investigate potential differences between Chinese and Korean participants' self-construal orientation; (iii) an eye-tracking paradigm to measure Chinese and Korean speakers' attention to background information and to focal objects; (iv) participants speaking non-East Asian languages that have a similar sentence structure to Korean, such as Turkish (Christophe et al., 2003); and (v) Korean-English bilingual participants in order to investigate whether bilinguals display either analytic attentional patterns or holistic patterns depending on the language condition. Additionally, future studies examining cross-cultural differences at the level of implicit cognition, as well as event-related fMRI studies investigating differences in neural activation patterns could reveal to what extent language affects elements of perception and cognition at large. To our knowledge, prior studies investigating holistic and analytic attentional patterns involved almost exclusively monolingual speakers (see Rayner et al., 2007 as the only exception). Thus, future studies exploring bilingual speakers' attention by manipulating language during scene perception might provide new insights helping to improve our understanding of how language contributes to the formation of the holistic attentional bias.

## Conclusion

The present research offers a first replication of Tajima and Duffield's (2012) findings and suggests new directions in research on holistic and analytic attentional patterns, such as cross-cultural research involving bilingual speakers. Moreover, it responds to critical views on the common practice of investigating cross-cultural differences by comparing Western participants with participants of a single East Asian culture (Henrich et al., 2010). As Varnum et al. (2010) point out, research comparing merely these two types of samples does not allow us to rule out alternative explanations for the emergence of attentional biases, such as the influence of language. Further research examining differences between cultures that have been assumed to be culturally similar might be a key to advance cross-cultural research and to push the boundaries of current theories. In addition, future studies might provide new insights which help us to understand the interrelation between language and culture, moving the long-standing debate on the language-culture relationship and effects on perception, information processing, and cognition in new directions. In this sense, there could be “new lines of work in the study of language that together constitute language's latest pendulum swing back into the world of culture” (Enfield, 2012, p. 166).

## Author contributions

AR conceived, developed, and conducted the experimental research. AR analyzed and interpreted the data. AR drafted the manuscript. BV and IG made direct and substantial intellectual contribution to the manuscript. All authors approved the work for publication.

### Conflict of interest statement

The authors declare that the research was conducted in the absence of any commercial or financial relationships that could be construed as a potential conflict of interest.
